# Supplementary material for: Rhythm Control in Patients with Heart Failure with Preserved Ejection Fraction: A Meta-Analysis
Source: J Clin Med. 2021 Sep 7;10(18):4038. doi: 10.3390/jcm10184038 (PMC8471283; doi:10.3390/jcm10184038)
Supplement: Supplementary file 1 [file jcm-10-04038-s001.zip › jcm-1366691-supplementary.pdf]

Search strategy

PUBMED

Search number	Query	Search Details	Results	Time
8	(((rhythm control) OR (rate control)) AND (atrial fibrillation)) AND ((heart failure with preserved ejection fraction) OR ("Heart Failure, Diastolic"[Mesh]))	((("rhythm"[All Fields] OR "rhythm s"[All Fields] OR "rhythms"[All Fields]) AND ("controling"[All Fields] OR "controllability"[All Fields] OR "controllable"[All Fields] OR "controllably"[All Fields] OR "controller"[All Fields] OR "controller s"[All Fields] OR "controllers"[All Fields] OR "controlling"[All Fields] OR "controls"[All Fields] OR "prevention and control"[MeSH Subheading] OR ("prevention"[All Fields] AND "control"[All Fields]) OR "prevention and control"[All Fields] OR "control"[All Fields] OR "control groups"[MeSH Terms] OR ("control"[All Fields] AND "groups"[All Fields]) OR "control groups"[All Fields])) OR (("j rehabil assist technol eng"[Journal] OR "rate"[All Fields]) AND ("controling"[All Fields] OR "controllability"[All Fields] OR "controllable"[All Fields] OR "controllably"[All Fields] OR "controller"[All Fields] OR "controller s"[All Fields] OR "controllers"[All Fields] OR "controlling"[All Fields] OR "controls"[All Fields] OR "prevention and control"[MeSH Subheading] OR ("prevention"[All Fields] AND "control"[All Fields]) OR "prevention and control"[All Fields] OR "control"[All Fields] OR "control groups"[MeSH Terms] OR ("control"[All Fields] AND "groups"[All Fields]) OR "control groups"[All Fields]))) AND ("atrial fibrillation"[MeSH Terms] OR ("atrial"[All Fields] AND "fibrillation"[All Fields]) OR "atrial fibrillation"[All Fields]) AND ((("heart failure"[MeSH Terms] OR ("heart"[All Fields] AND "failure"[All Fields]) OR "heart failure"[All Fields]) AND ("preservation, biological"[MeSH Terms] OR ("preservation"[All Fields] AND "biological"[All Fields]) OR "biological preservation"[All Fields] OR "preservation"[All Fields] OR "preserved"[All Fields] OR "preservations"[All Fields] OR "preserve"[All Fields] OR "preserves"[All Fields] OR "preserving"[All Fields]) AND ("eject"[All Fields] OR "ejected"[All Fields] OR "ejecting"[All Fields] OR "ejection"[All Fields] OR "ejectional"[All Fields] OR "ejections"[All Fields] OR "ejects"[All Fields]) AND ("dose fractionation, radiation"[MeSH Terms] OR ("dose"[All Fields] AND "fractionation"[All Fields] AND "radiation"[All Fields]) OR "radiation dose fractionation"[All Fields] OR "fractionation"[All Fields] OR "chemical fractionation"[MeSH Terms] OR ("chemical"[All Fields] AND "fractionation"[All Fields]) OR "chemical fractionation"[All Fields] OR "fraction"[All Fields] OR "fraction s"[All Fields] OR "fractionate"[All Fields] OR "fractionated"[All Fields] OR "fractionates"[All Fields] OR "fractionating"[All Fields] OR "fractionationed"[All Fields] OR "fractionations"[All Fields] OR "fractionator"[All Fields] OR "fractionators"[All Fields] OR "fractioned"[All Fields] OR "fractioning"[All Fields] OR "fractionized"[All Fields] OR "fractions"[All Fields])) OR "heart failure, diastolic"[MeSH Terms])	81	20:54:16

7	(rhythm control) OR (rate control)	(("rhythm"[All Fields] OR "rhythm s"[All Fields] OR "rhythms"[All Fields]) AND ("controling"[All Fields] OR "controllability"[All Fields] OR "controllable"[All Fields] OR "controllably"[All Fields] OR "controller"[All Fields] OR "controller s"[All Fields] OR "controllers"[All Fields] OR "controlling"[All Fields] OR "controls"[All Fields] OR "prevention and control"[MeSH Subheading] OR ("prevention"[All Fields] AND "control"[All Fields]) OR "prevention and control"[All Fields] OR "control"[All Fields] OR "control groups"[MeSH Terms] OR ("control"[All Fields] AND "groups"[All Fields]) OR "control groups"[All Fields])) OR (("j rehabil assist technol eng"[Journal] OR "rate"[All Fields]) AND ("controling"[All Fields] OR "controllability"[All Fields] OR "controllable"[All Fields] OR "controllably"[All Fields] OR "controller"[All Fields] OR "controller s"[All Fields] OR "controllers"[All Fields] OR "controlling"[All Fields] OR "controls"[All Fields] OR "prevention and control"[MeSH Subheading] OR ("prevention"[All Fields] AND "control"[All Fields]) OR "prevention and control"[All Fields] OR "control"[All Fields] OR "control groups"[MeSH Terms] OR ("control"[All Fields] AND "groups"[All Fields]) OR "control groups"[All Fields]))	563,107	20:48:03
6	(heart failure with preserved ejection fraction) OR ("Heart Failure, Diastolic"[Mesh])	(("heart failure"[MeSH Terms] OR ("heart"[All Fields] AND "failure"[All Fields]) OR "heart failure"[All Fields]) AND ("preservation, biological"[MeSH Terms] OR ("preservation"[All Fields] AND "biological"[All Fields]) OR "biological preservation"[All Fields] OR "preservation"[All Fields] OR "preserved"[All Fields] OR "preservations"[All Fields] OR "preserve"[All Fields] OR "preserves"[All Fields] OR "preserving"[All Fields]) AND ("eject"[All Fields] OR "ejected"[All Fields] OR "ejecting"[All Fields] OR "ejection"[All Fields] OR "ejectional"[All Fields] OR "ejections"[All Fields] OR "ejects"[All Fields]) AND ("dose fractionation, radiation"[MeSH Terms] OR ("dose"[All Fields] AND "fractionation"[All Fields] AND "radiation"[All Fields]) OR "radiation dose fractionation"[All Fields] OR "fractionation"[All Fields] OR "chemical fractionation"[MeSH Terms] OR ("chemical"[All Fields] AND "fractionation"[All Fields]) OR "chemical fractionation"[All Fields] OR "fraction"[All Fields] OR "fraction s"[All Fields] OR "fractionate"[All Fields] OR "fractionated"[All Fields] OR "fractionates"[All Fields] OR "fractionating"[All Fields] OR "fractionationed"[All Fields] OR "fractionations"[All Fields] OR "fractionator"[All Fields] OR "fractionators"[All Fields] OR "fractioned"[All Fields] OR "fractioning"[All Fields] OR "fractionized"[All Fields] OR "fractions"[All Fields])) OR "heart failure, diastolic"[MeSH Terms]	6,827	20:47:39
5	rhythm control	("rhythm"[All Fields] OR "rhythm s"[All Fields] OR "rhythms"[All Fields]) AND ("controling"[All Fields] OR "controllability"[All Fields] OR "controllable"[All Fields] OR "controllably"[All Fields] OR "controller"[All Fields] OR "controller s"[All Fields] OR "controllers"[All Fields] OR "controlling"[All Fields] OR "controls"[All Fields] OR "prevention and control"[MeSH Subheading] OR ("prevention"[All Fields] AND "control"[All Fields]) OR "prevention and control"[All Fields] OR "control"[All Fields] OR "control groups"[MeSH Terms] OR ("control"[All Fields] AND "groups"[All Fields]) OR "control groups"[All Fields])	37,052	20:47:06

4	rate control	("j rehabil assist technol eng"[Journal] OR "rate"[All Fields]) AND ("controling"[All Fields] OR "controllability"[All Fields] OR "controllable"[All Fields] OR "controllably"[All Fields] OR "controller"[All Fields] OR "controller s"[All Fields] OR "controllers"[All Fields] OR "controlling"[All Fields] OR "controls"[All Fields] OR "prevention and control"[MeSH Subheading] OR ("prevention"[All Fields] AND "control"[All Fields]) OR "prevention and control"[All Fields] OR "control"[All Fields] OR "control groups"[MeSH Terms] OR ("control"[All Fields] AND "groups"[All Fields]) OR "control groups"[All Fields])	534,521	20:46:59
3	atrial fibrillation	"atrial fibrillation"[MeSH Terms] OR ("atrial"[All Fields] AND "fibrillation"[All Fields]) OR "atrial fibrillation"[All Fields]	86,855	20:46:50
2	"Heart Failure, Diastolic"[Mesh]	"heart failure, diastolic"[MeSH Terms]	777	20:46:11
1	heart failure with preserved ejection fraction	("heart failure"[MeSH Terms] OR ("heart"[All Fields] AND "failure"[All Fields]) OR "heart failure"[All Fields]) AND ("preservation, biological"[MeSH Terms] OR ("preservation"[All Fields] AND "biological"[All Fields]) OR "biological preservation"[All Fields] OR "preservation"[All Fields] OR "preserved"[All Fields] OR "preservations"[All Fields] OR "preserve"[All Fields] OR "preserves"[All Fields] OR "preserving"[All Fields]) AND ("eject"[All Fields] OR "ejected"[All Fields] OR "ejecting"[All Fields] OR "ejection"[All Fields] OR "ejectional"[All Fields] OR "ejections"[All Fields] OR "ejects"[All Fields]) AND ("dose fractionation, radiation"[MeSH Terms] OR ("dose"[All Fields] AND "fractionation"[All Fields] AND "radiation"[All Fields]) OR "radiation dose fractionation"[All Fields] OR "fractionation"[All Fields] OR "chemical fractionation"[MeSH Terms] OR ("chemical"[All Fields] AND "fractionation"[All Fields]) OR "chemical fractionation"[All Fields] OR "fraction"[All Fields] OR "fraction s"[All Fields] OR "fractionate"[All Fields] OR "fractionated"[All Fields] OR "fractionates"[All Fields] OR "fractionating"[All Fields] OR "fractionationed"[All Fields] OR "fractionations"[All Fields] OR "fractionator"[All Fields] OR "fractionators"[All Fields] OR "fractioned"[All Fields] OR "fractioning"[All Fields] OR "fractionized"[All Fields] OR "fractions"[All Fields])	6,252	20:45:25

EMBASE

**No.**	**Query**	**Results**
**#2**	(('**heart**'/exp OR **heart**) AND ('**failure**'/exp OR **failure**) AND **with** AND **preserved** AND **ejection** AND **fraction** OR '**heart failure, diastolic**'/exp OR '**heart failure, diastolic**' OR **hfpef**) AND (('**rhythm**'/exp OR **rhythm**) AND ('**control**'/exp OR **control**) OR (**rate** AND ('**control**'/exp OR **control**))) AND **atrial** AND ('**fibrillation**'/exp OR **fibrillation**) AND [humans]/lim	**211**

OVID

Query	Searches	Results
1	Heart failure. Diastolic	777
2	Atrial fibrillation	56977
3	rate control.mp.	3367
4	rhythm control.mp.	1705
5	heart failure with preserved ejection fraction.mp.	3048
6	HFPEF.mp.	2793
7	1 OR 5 OR 6	4675
8	3 OR 4	4346
9	2 AND 7 AND 8	12

Cochrane

Query	Searches	Results
1	Diastolic heart failure	4094
2	Atrial fibrillation	12976
3	rate control	105066
4	rhythm control	4292
5	heart failure with preserved ejection fraction	1286
6	HFPEF	721
7	1 OR 5 OR 6	4996
8	3 OR 4	107539
9	2 AND 7 AND 8	125

Figure S1

Forrest plot

HF admission

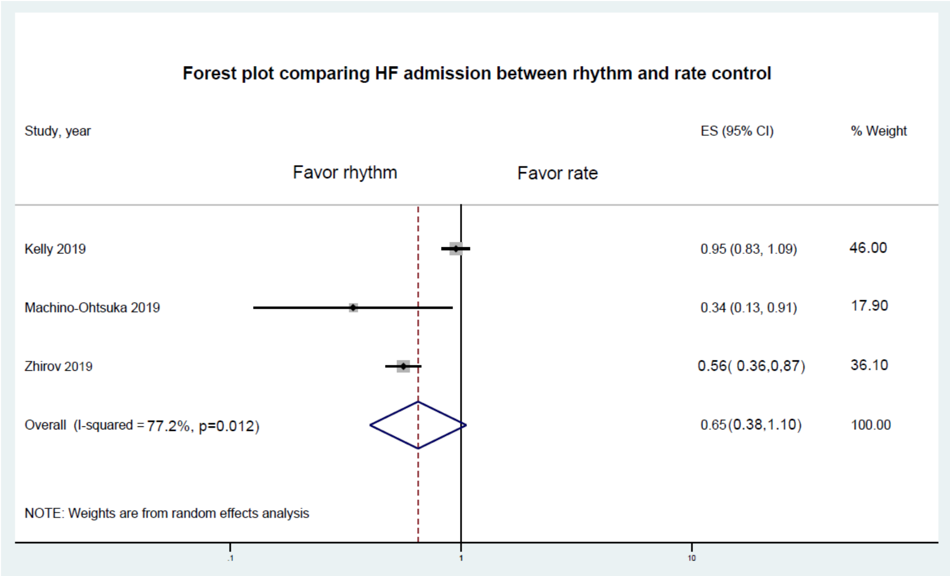

CV mortality/TIA and stroke

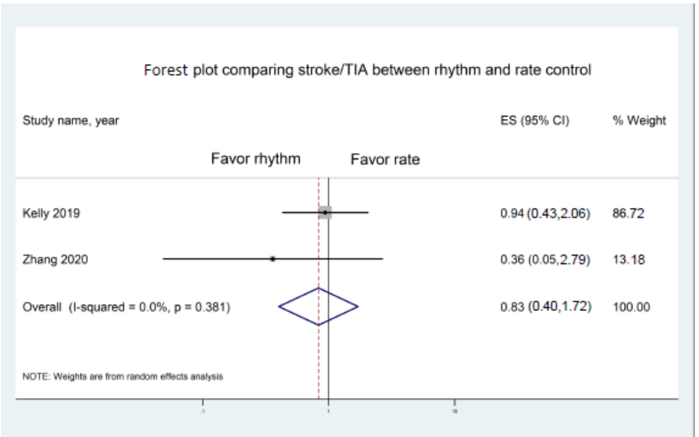

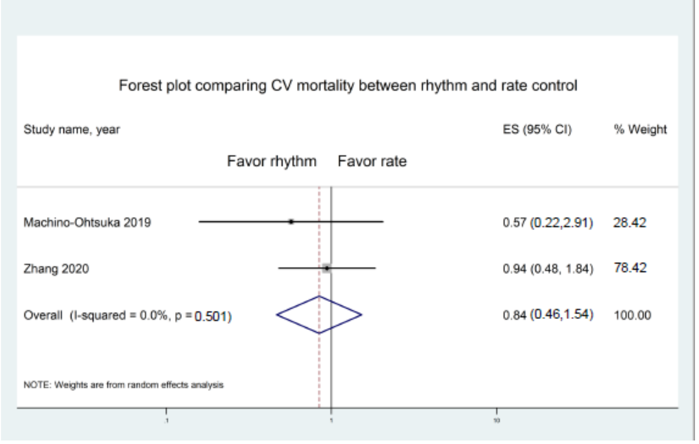

Figure S2

Funnel plot

Long term mortality

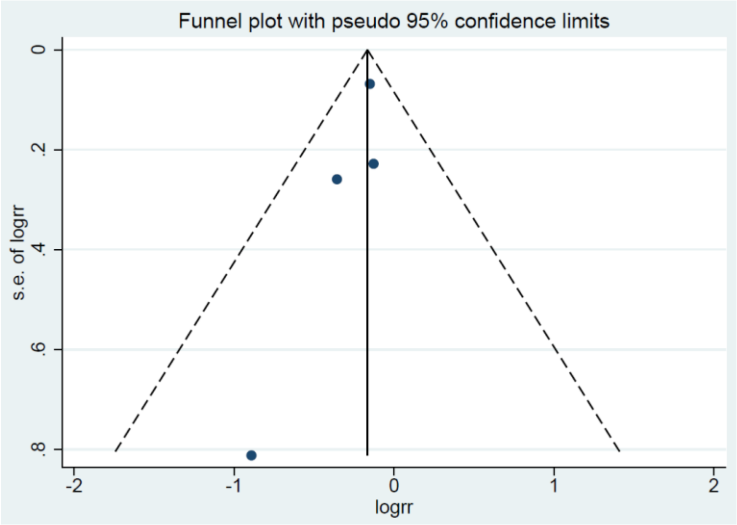

HF admission

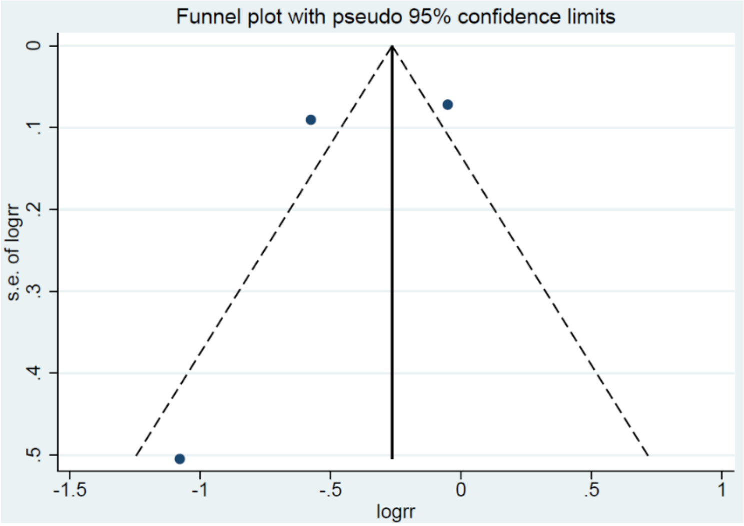

CV mortality

Stroke/TIA

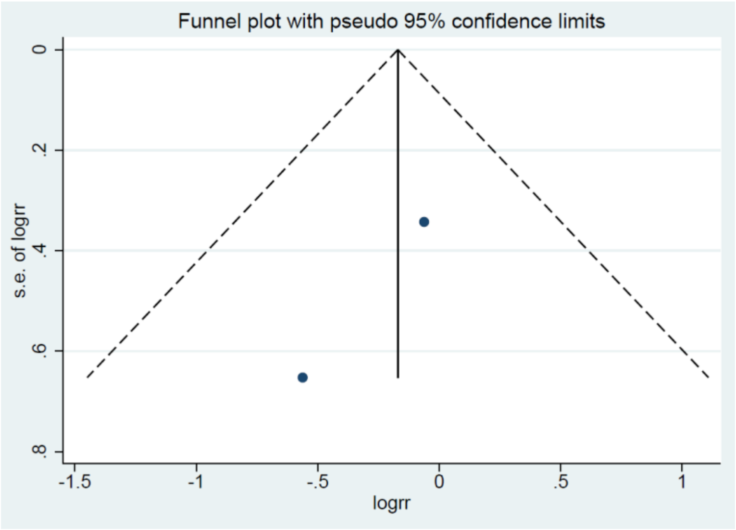

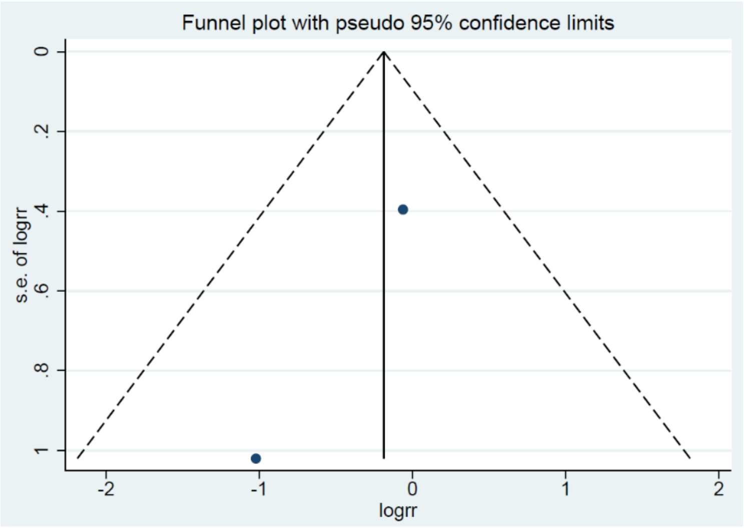

Figure S3

Sensitivity analysis

Long-term mortality

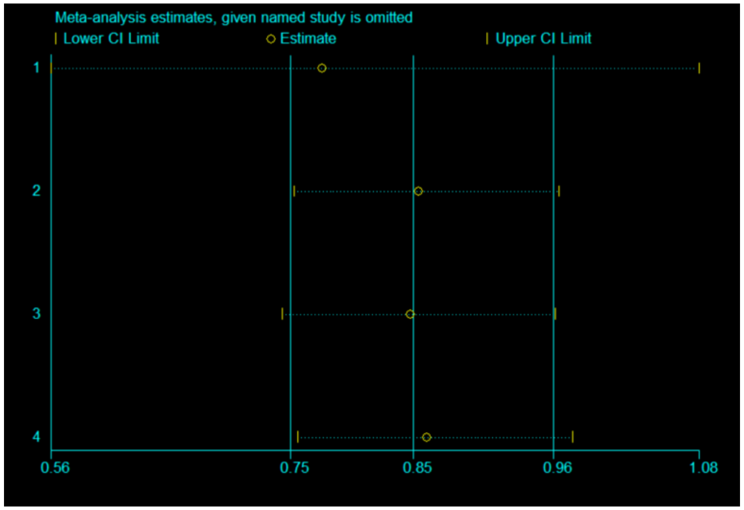

HF admission

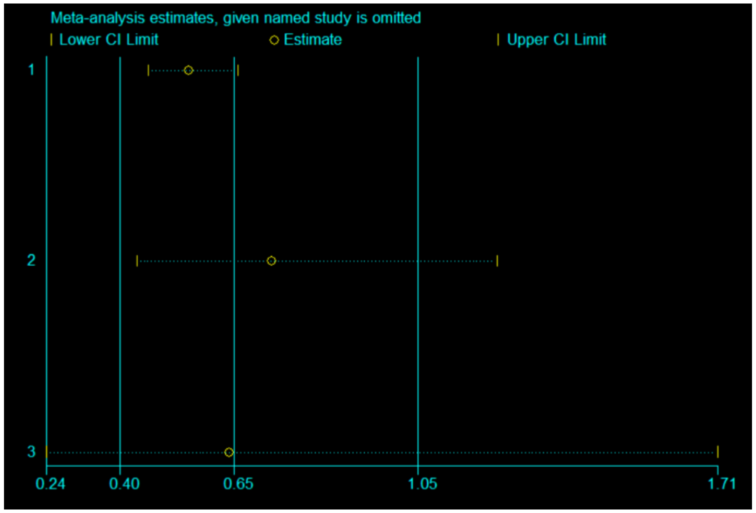

CV mortality

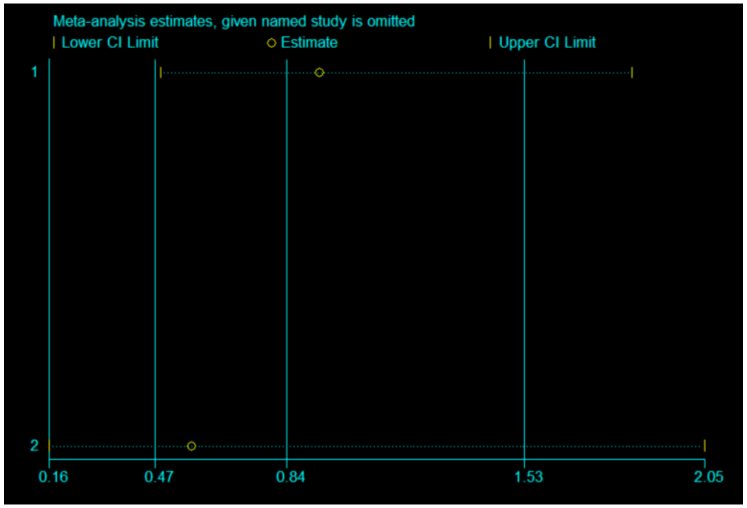

Stroke/TIA

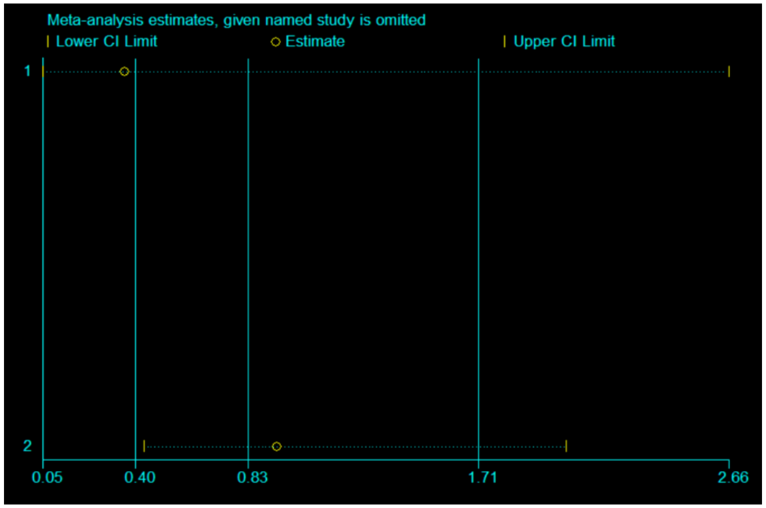

Table S1: Newcastle-Ottawa quality assessment scale of included studies in meta-analysis

Study	Selection				Comparability (Confounding)	Outcome			Total
	Representative of exposed cohort	Selection of the non-exposed cohort	Ascertainment of exposure	Endpoint not presenting at start		Assessment of Outcome	Follow-up duration	Adequacy follow-up	
Kelly 2019	*	*	*	*	*	*	*	*	8
Machino-Ohtsuka 2019	*	*	*	*	**	*	*	*	9
Slee 2019	*	*	*	*	*	*	*	*	8
Zhang 2020	*	*	*	*	*	*	*	*	9
Zhirov 2019	*	*	*	*		*	*	*	7
